# Factors Influencing the Reputation of Assistive Technology Resources Center: An Example from Yunlin County, Taiwan

**DOI:** 10.3390/healthcare10020243

**Published:** 2022-01-27

**Authors:** Tsen-Yao Chang, Shao-Wei Huang

**Affiliations:** 1 Department of Creative Design, National Yunlin University of Science and Technology, Douliou 640, Taiwan; 2 Graduate School of Design, National Yunlin University of Science and Technology, Douliou 640, Taiwan; d10530017@gemail.yuntech.edu.tw

**Keywords:** assistive technology services, assistive technology resources center, public institution reputation

## Abstract

By the end of 2020, a total of 34 multifunctional Assistive Technology Resources Centers (ATR centers) were set up in 22 counties and cities in Taiwan. This study examines the perceptions of the users and their caregivers of the government-established ATR centers in Taiwan and examines the impact on the reputation of the public institution. The research framework and hypotheses were developed by examining the factors of “service convenience”, “center-related factors”, “justice”, and “perceived value” and using “perceived value” as a mediating variable. The data were collected through a questionnaire survey, and the structural equation model was used to test the model and verify the hypotheses. Research data was collected in various townships in Yunlin County A total of 320 questionnaires were collected. Of these respondents, 22% were aged 51–60. All the research hypotheses were positively and significantly verified. Of these, justice was the most important factor affecting the value of the ATR center’s services compared to convenience and center-related factors. Of convenience, service value and justice, service value was the most important factor affecting the perceived reputation of the public institution. According to the findings of this study, it is beyond expectation that the convenience of ATR is not the main factor influencing the service value, but rather the perceived justice is the most important factor. Therefore, ATR should be prioritized from the perspective of the service recipient, especially the perceived justice of the service, in order to best enhance the value of the service and improve the reputation of the public institution.

## 1. Introduction

According to the World Health Organization [1], the number of people over 60 years of age will double from 2015 to 2050, significantly increasing from 12 percent to 22 percent of the total population. At the same time, Taiwan is gradually moving into an aged society. By January 2021, there will be 3.804 million people over the age of 65, accounting for 16.2% of the total population, and will enter a super-aged society in 2025. The proportion of the elderly population is expected to be as high as 41.2% by 2065 [2]. In addition, there are 1,197,887 people with disabilities in Taiwan, accounting for about 5% of the total population, of which more than 70% are over 50 years old.

Taiwan has officially entered an aged society. With the overall demographic structure of the population rapidly trending towards very old age, and the number of people with disabilities reaching over 1 million, Taiwan is facing a double dilemma of a huge increase in the number of people in need of assistive devices and a shortage of caregivers. The use of assistive devices can reduce the caregiver’s burden, enhance the care recipient’s ability to move independently, and delay disability. Therefore, the government need how to provide professionals to consider, comprehensive and convenient assistive device services to the care recipients and their caregivers.

Especially, assistive technology includes assistive technology devices and assistive technology services [3]. The use of assistive devices can help people with disabilities to overcome physical barriers in order to enhance their functions; therefore, assistive devices may be used by people with physical and mental disabilities, elderly people with disabilities, or children with special education needs. However, the use of assistive devices usually requires special service which includes consultation, evaluation before obtaining the assistive devices or training and maintenance services after use, etc. Each service can be a tangible or intangible touchpoint. For example, to find a service location or to apply for aids, etc. Through human resources, hardware, software, and information, the service providers can gradually shape the experience of each user. Therefore, whether it is the service process, the space design of the service experience, or the feeling of the equipment experience, all will affect the feeling of being served, and then produce the evaluation effect.

In Taiwan, the Disability Welfare Act was enacted in 1980 to provide subsidies for assistive devices, such as wheelchairs and hearing aids, for the people with disabilities. In 2012, the regulations for the people with disabilities were amended [4]. The county and city governments should set up “assistive technology resource centers” to provide assistive device services, including assistive devices demonstration, consultation, evaluation, and trial use, repairing, recycling, and training, etc., to provide professional services related to the use of assistive devices for the people with disabilities and the disabled elderly. The center is equipped with professional staff such as physiotherapists, occupational therapists, social workers, hearing specialists and maintenance technicians. By the end of 2020, a total of 34 multifunctional Assistive Technology Resources Centers (ATR centers) were set up in 22 counties and cities in Taiwan, operated by local governments or commissioned by Non-Governmental Organizations (NGOs).

At the same time, too enhance the accessibility of services, since 2018, each county and city government has started to set up “assistive technology service bases” and “assistive technology convenience stations” in conjunction with the existing publicly-owned venues within their jurisdictions. Through the establishment of these three levels of service resources (resource center, service base and convenience stations), ATR centers can provide appropriate aids services to people who in need.

In Taiwan, the government has considered the need to provide appropriate assistive device services for the people with disabilities and the disabled elderly in addition to the provision of assistive device subsidies and has therefore planned to promote related policies simultaneously with a complete service framework of “assistive device subsidies” and “assistive device services” [5]. In the early stage of resource center establishment, the main direction was to establish the service system, cultivate talents for assistive device service, and enrich the basic service energy, etc. Recently, in view of the advent of the aged society, the demand for assistive devices in counties and municipalities has increased rapidly [6]. The government is expected to provide better convenience services to the public.

However, due to the uneven resources and local ecological differences, such as demographics, geography, medical habits, and even accessibility, there is no way to know whether the assistive services planned by the government will meet the expectations and needs of the public. Therefore, when users have the need for assistive device services, how to find suitable assistive device services with the existing resources, and how to provide more satisfactory services to enhance the reputation of the government while the government is setting up resources for assistive device services, are the issues that governments at all levels are urgently facing in the current aged society.

Whether it is a physically challenged person or a disabled elderly person, in the overall process of assistive device service delivery, the center will identify the needs and define the problems of the service cases, and then provide appropriate assistive devices suggestions after assessment. In addition, after the use of assistive aids, it is necessary to provide follow-up training [7]. At the same time, the service process also includes obtaining relevant subsidies to ensure that users can receive the suitable aids [8].

Long-term care insurance system in Japanese began in 2000 and is designed primarily for people with long-term care needs. This also includes Assistive Devices Payment System, which includes 13 kinds of subsidies for assistive device rental, 5 kinds of subsidies for assistive device purchases, and payments for residential renovation [9]. This system helps the elderly aged 65 and above and the disabled aged 40–64 to live independently, delaying their functional deterioration or reducing the burden on caregivers [10]. At the same time, the Japanese government also provides assistive device services for long-term care clients through the welfare device leasing industry of the nursing care insurance.

The people with disabilities are unable to adapt to the workplace environment due to physical or psychological impairments. Job accommodation can effectively open up employment opportunities for people with physical disabilities and eliminate barriers to work [11]. Through a complete job analysis and assessment of employees’ potential abilities, the center can better understand their employability needs. Assistive technology is used to enhance case’s abilities and help employees with physical disabilities stabilize their employment.

This study will examine the perceptions of users of assistive devices or their caregivers regarding the use of ATR center-related services and examine the influencing factors. After the literature review, convenience of service, factors related to the ATR center, justice and perceived value were identified as independent variables, among which perceived value also played the role of a mediating variable. The objectives of this study are to understand the impact on the prestige of the public institution after the users of assistive devices received services and the importance of convenience of service, factors related to the ATR center, justice and perceived value from the government-appointed Yunlin ATR center. Service convenience, central related factors and justice should positively and significantly affect perceived value, and the perceived value of the service recipient will affect their perception of the reputation of the public institution. In the future, when the demand for assistive devices is increasing drastically in the aged society, it is recommended that government departments should intervene through policy or service design to build a better assistive device service system, so that users can easily obtain the appropriate assistive device services and provide better service experience to the public.

## 2. Literature Review

### 2.1. Taiwan Assistive Technology Resource Centers (ATR Center)

**Figure 1:**
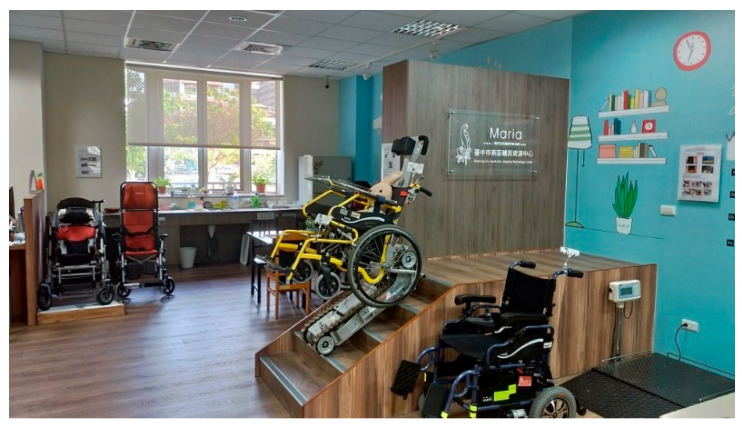
Taichung City South District Assistive Resource Center (the image was shot by the authors).

**Figure 2:**
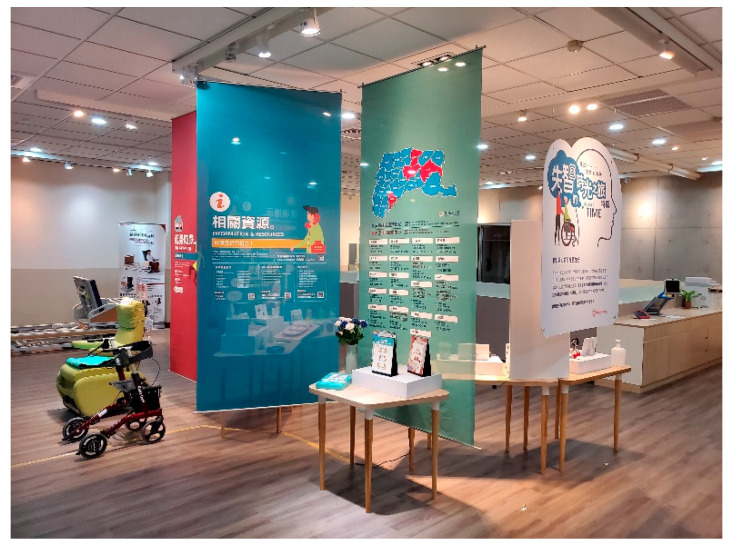
Assistive Resource Center in Yunlin County (the image was shot by the authors).

In 1980, Taiwan enacted the “Disability Welfare Act” to subsidize assistive aids, such as wheelchairs, hearing aids and eyeglasses, needed by people with physical disabilities. After years of revising the laws and regulations related to people with disabilities, the Ministry of the Interior amended the “Law for the Protection of People with disabilities” [12] in 1997 and began to set up ATR centers in every county and city to provide assistive device related services for the physically challenged, such as professional consultation, assistive device evaluation, assistive device repair, recycling, demonstration, etc. [13]. In 2012, the Law on the Protection of the Rights of Persons with Disabilities [14] was amended to establish regulations on the services provided by the ATR centers in each county and city, including the business of the assistive aids centers, the function and size of the space, and even the types of related professional manpower. Figure 1 and Figure 2 demonstrate ATR center fields.

**Figure 3:**
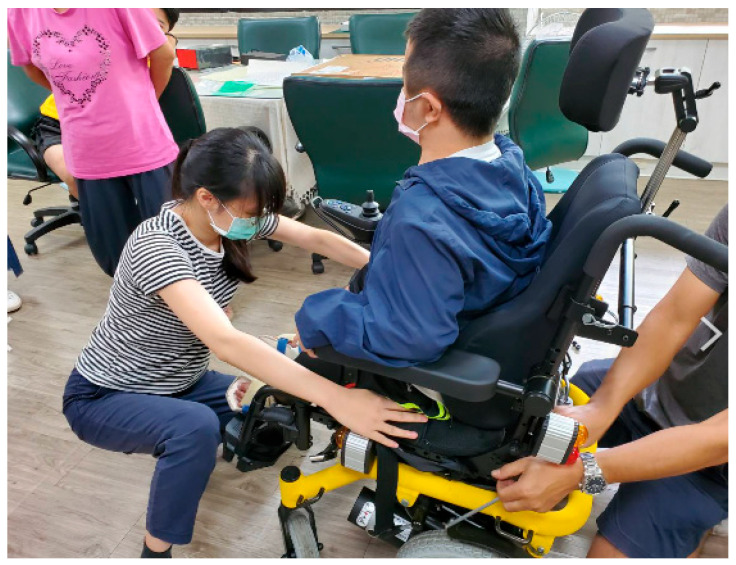
Assistive aid assessment at school (the image was shot by the authors).

As of the end of 2020, there are 34 assistive resource centers in counties and cities across the country, run by local governments or commissioned by NGOs, providing services for the people with disabilities, the disabled elderly, special education students, and physically challenged workers as Figure 3 displayed.

In addition, due to the advent of an aging society, the demand for assistive device services has increased significantly. In order to improve the overall service quality and enhance the service details, public institutions are paying more attention to the users’ feelings about the service contact points and related service processes, hoping to give assistive device users a better service experience and thus generate a better satisfaction level.

### 2.2. Service Convenience

Consumers’ perception of service convenience is mainly focused on the time and effort spent to obtain the service, which is divided into five categories, namely, the convenience of decision making, obtaining the service, transaction, profit, and after-sale service [15]. It is also affected by the characteristics of the service, the firm-related factors of the selling company or the service provider, and individual consumer differences. In terms of the sales company or the service provider itself, the overall service environment, the message to the consumer, and the company brand may all affect the convenience of the service.

Chang and Polonsky [16] have examined the impact of multiple service convenience on behavioral intentions of Taiwanese consumers in health clubs. The study found that the increase in satisfaction with the service, especially if they feel the convenience of profit (benefit convenience) and after-sales service (postbenefit convenience), will generate positive behavioral intentions, for example, repeat consumption and word-of-mouth. It is also possible to find broader applications for the impact of services by looking at different types of service convenience.

In addition, Chen et al. [17], in their study on the use of online ticket booking systems by affordable airline travelers, found that the higher the technology readiness of a company, the more positive the impact on the company’s reputation and convenience of service. The study collected a total of 422 questionnaires from AirAsia X passengers and found that ease of decision making was the most important aspect of service convenience. If passengers can use the 24 h ticketing service through the app during their journey to buy tickets or get the latest news, or to inquire about promotions or connection information, it will help passengers improve their reputation and satisfaction with the company. Therefore, this study concludes that the perceived convenience of the services provided to the clients at the ATR Center will affect the perceived value of the services.

### 2.3. Perceived Value

Perceived value is the overall subjective evaluation of the trade-off between what a consumer pays and what he or she gets when acquiring a good or service [18]. Zeithaml [19] divided it into four categories, including value is low price, value is whatever I want in a product, the trade-off between the “price” I pay and the “quality” I get, and everything related to “pay” and “get”.

Pham et al. [20] found that perceived value is an important key to satisfaction and behavioral intent in their study of repurchase rates for online shopping in Vietnam. Many studies have also found that perceived value may be a better predictor of repurchase intentions than satisfaction or quality, indicating that perceived value has a positive effect on repurchase intentions; and perceived value is positively correlated with factors such as purchase intentions.

In addition, in the mobile industry, the challenge of retaining existing users has been a constant one. Therefore, Chun-Mei Chen [21] investigated how service quality, brand image and perceived value affect customer satisfaction and loyalty, which in turn affects customer churn. It was found that perceived value has the greatest impact on customer satisfaction, while brand image has the greatest impact on loyalty, and perceived value has less impact. It is also suggested that mobile communication operators should focus on strengthening their customers’ perceived value to ensure consumers’ satisfaction with their customers.

Moreover, for the popularity of low-cost fitness business activities in Spain, the results of a study on customer loyalty show that convenience of service has a significant impact on perceived value [22]. Behavioral intentions for future re-visits are most strongly associated with loyalty, followed by satisfaction and convenience of service.

In addition, in a study conducted by Kuo-Chien Chang [23] et al. on Chinese restaurant chains, customer satisfaction was strongly influenced by the convenience of service. Customer loyalty is positively influenced by satisfaction, while customer satisfaction is indirectly influenced by perceived service value and service convenience. It is clear that convenience of service has a clear relevance to the perceived value of consumers. Therefore, this study concluded that the service convenience perceived by the clients at the ATR centers, such as the availability of the assistive aid center, the convenience of contact, the business hours, the geographical location, and the collection of relevant information, would positively and significantly affect their perceived value, and proposed the following research hypotheses.

**Hypothesis** **1** **(H1).** *The service convenience of the ATR center will positively and significantly affect the user’s perceived value*.

### 2.4. The ATR Center Related Factors

Company or unit factors influence consumer perceptions of service convenience [15], including service environment, consumer information, company brand, and service system design. Bitner [24] explored the impact of the physical environment on customer and employee behavior, and its relevance to management, suggesting that consumers are affected by the physical environment to the extent that they change their behavior and judgments.

Bitner [24] explored the impact of the physical environment on customer and employee behavior, and its relevance to management, suggesting that consumers are affected by the physical environment to the extent that they change their behavior and judgments. Baker and Cameron [25] argued that customers’ subjective perceptions of waiting time in a store are influenced by environmental elements such as temperature and music, or design elements such as lighting, furniture arrangement, or space layout, or even social elements such as staff-customer interaction. In a study of information delivered by the online booking site Agoda.com (accessed on 17 December 2021), it was found that visual information (e.g., photos of rooms or facilities) had a positive effect on consumer behavior when given to consumers through the site as opposed to text [26]. This part can be rated by online rating and accommodation experience.

In addition, in terms of branding factors, Kuan-Yin Lee [27] and others found a positive association between branding and convenience of service through retail services such as Starbucks, McDonald’s, and Warner Viejo. At the same time, service convenience also indirectly affects brand loyalty positively through satisfaction. In addition, Berry et al. [15] suggest that the time and effort of consumers in accessing services can be managed through service system design; for example, by improving mobility, customers can find the right products more efficiently and increase the overall convenience of services.

In conclusion, for the ATR centers, the study concluded that the center-related factors would affect the value of the service recipients after receiving the service, including the internal activity space, the overall equipment, and the professionalism, reliability, and intimacy of the center’s brand to the public. The research hypotheses are as follows:

**Hypothesis** **2** **(H2).** *The factors related to the ATR center will positively and significantly affect the user’s perceived value*.

### 2.5. Justice

Skarlicki and Folger [28] studied the connection between organizational justice and organizational retaliation behavior to investigate whether unfair treatment of employees leads to anger, dissatisfaction, and specific behaviors, and even retaliatory behavior in the workplace. They also classified justice into three categories: distributive, procedural, and interactional. Then, scholars such as Hossain (2021) explored the interrelationship between service justice, service quality, social influence, and corporate image regarding service satisfaction and loyalty in the retail bank industry. As mentioned in the report, service justice and service quality have a significant impact on service satisfaction and customer loyalty.

Moreover, Chao-Min, Chao-Sheng, and Hae-Ching et al. [29] investigated learners’ intention to use Web-based learning and found that distributive and interactive equity had a significant positive effect on Web-based learning satisfaction. The justice of the procedure and the satisfaction with e-learning play an important role in continued use [30].

Chao-Min, Chao-Sheng, and Hae-Ching et al. [29], in their study of learners’ intention to use Web-based learning, it was found that distributive justice and interactive justice had a significant positive effect on e-learning satisfaction. The procedure justice and the satisfaction with e-learning play an important role in continued use. In addition, previous studies have shown that interactional fairness has a positive impact on customer satisfaction, customer loyalty, and customer revisit intention. [31,32].

Some studies indicate that distributional justice has a positive impact on job satisfaction [33]. In addition, if the justice of distribution can be improved, it will lead to more consumers’ repurchase intention [34]. Mwesiumo and Halpern [35] found that inter-firm communication that generates partner acquiescence has a positive effect on firm operations, with distributional equity having the greatest impact, even greater than irreplaceability, and reducing conflict between exchanges.

Moreover, Lind and Tyler [33] proposed a group-value theory, which suggests that if a group process is perceived to be justice, members of the group will naturally follow the group’s behavior. That is, if the process that produces the outcome is justice, one will be satisfied with the outcome regardless of whether the outcome is fair or not. This theory is also based on group norms and relations rather than on social exchange theory. In addition, Maxham and Netemeyer [32] also argue that a better understanding of procedural justice by customers would help to increase long-term satisfaction between buyers and sellers. In addition, Maxham and Netemeyer [32] also argued that a better understanding of procedural justice by customers would help to increase long-term satisfaction between buyers and sellers.

A study of customer behavioral intentions in the catering industry found that quality service enhances operational success, and that if customers feel justified in the service delivery process, it enhances satisfaction and increases the likelihood of repeat purchases [36]. Moreover, another study on the behavioral intentions of customers of the Cantonese Yum Cha service found that interaction and procedural justice had a significant impact on negative word-of-mouth in the event of service failure severity. In addition, perceived righteousness has been found to produce psychological contract violations on consumer behavioral intentions through the mediation of emotions [37,38]. In addition, Diana’s study [39] of healthcare organizations, which surveyed over 400 patients in the US and Spain, found that patient perceptions of justice had a significant impact on organizational satisfaction and trust in services. Furthermore, Yang Liu [40] conducted a study on the effect of justice on customer loyalty in automotive recall services. The study found that distribution and procedures justice had a positive effect on trust, indicating that justice had a significant impact on customer behavior.

In summary, justice has a positive effect on perceived value, especially for clients who are relatively disadvantaged groups and have a higher need to be treated fairly in the service process [41]. Therefore, this study hypothesizes that justice has a positive and significant effect on perceived value and proposes the following hypothesis:

**Hypothesis** **3** **(H3).** *The perception of justice of the ATR center’s services will positively and significantly affect the user’s perceived value*.

### 2.6. Public Institution Reputation

The reputation of an organization plays a very important role in the overall development of the organization. The reputation will affect the behavior intentions of customers and reflect their views on the quality of service [42]. Seigneur [43] argued that reputation is the result of perceptions of other people’s assessment of quality, characteristics or competence. Moreover, it is usually the overall subjective assessment of the value, called reputation, that is obtained without ever interacting with these people. A good reputation can help customers decide whether to buy a service before they understand the quality of the service.

For service-oriented companies, maintaining and expanding the relationship with customers is the main competitive advantage that differentiates them from other companies. From the study of Milan et al. [44], it is known that customer’s perceived value has a very significant impact on the reputation of the service provider. Moreover, service providers can enhance their competitiveness through a good company reputation, which in turn enhances the overall value of the company. Moreover, in a study on HRM and company reputation, Koys [45] pointed out that the goal of justice treatment of employees and company reputation are correlated. The overall reputation of the company will be enhanced if fair treatment is accepted.

From the study of the intention of mobile application software (APP) usage in hotel chains, the convenience of APP service has a positive impact on corporate reputation [46] The study by Chen et al. [17] also confirmed that corporate reputation is correlated with service convenience. Moreover, Casimiro Almeida and Coelho [47] found a correlation between perceived value and prestige in a study conducted on corporate reputation and its impact on brand equity. In a study of corporate reputation and its impact on brand equity, it was found that perceived value and reputation are correlated. A study by Bontis, et al. [48] also found that customer satisfaction enhances reputation in a service environment, and perceived value positively influences reputation through customer satisfaction.

In recent years, public institutions have been paying more and more attention to people’s opinions, hoping to understand the actual feedback from the public as a reference for improving governance. Therefore, public institutions have also begun to enhance the public’s reputation of the government by optimizing related services.

In this study, we consider the convenience, perceived value, and justice of the services provided by the public institution to the people. We believe that it has a positive and significant impact on the reputation of the service provider. In summary, service convenience, perceived value and justice have a positive effect on the reputation of public institutions. For users of assistive center services, the higher the perceived degree of service convenience, perceived value and justice, the higher the reputation of public institutions. Therefore, this study hypothesizes that service convenience, perceived value and justice have a positive and significant effect on the reputation of public institutions and proposes the following research hypotheses:

**Hypothesis** **4** **(H4).** *The perceived service convenience of the ATR center’s services will positively and significantly affect the reputation of the public institution*.

**Hypothesis** **5** **(H5).** *The perceived justice of the ATR center’s services will positively and significantly affect the reputation of the public institution*.

**Hypothesis** **6** **(H6).** *The perceived value of the ATR center’s services will positively and significantly affect the reputation of the public institution*.

### 2.7. Mediation Effect

When a third variable is used to produce the result between the independent variable and the dependent variable, that is, the independent variable affects the mediating variable and the mediating variable affects the dependent variable, which is called the mediation effect [49]. Caruana and Ewing [50] studied two online stores and found that corporate reputation has a direct impact on consumer loyalty online, while perceived value plays an important mediating role in the impact of online loyalty. For example, corporate sales usually attract consumers by reducing prices or improving the quality of goods; however, a study [51] showed that consumers are more willing to pay higher prices for the same quality of goods when the retail store has a higher reputation, which is known as the price-over-quality effect. In other words, through the intermediary of perceived value, consumers’ reputation of the retail store is enhanced, and the goods are recognized as being of higher quality, which can reduce the store’s means of promotion through price reduction.

Summarizing the above studies, service convenience, ATR center-related factors and justice have positive effects on perceived value, and perceived value also has positive effects on the reputation of public institution. The higher the perceived value for users of assistive devices, the better their perception of the public sector’s prestige. Therefore, this study hypothesizes that perceived value has a positive and significant effect on the reputation of public institutions. The following research hypotheses are proposed.

**Hypothesis** **7** **(H7).** *The perceived value of the ATR center’s services has a mediation effect between perceived service convenience and the reputation of the public institution*.

**Hypothesis** **8** **(H8).** *The perceived value of the ATR center’s services has a mediation effect between the factors related to the ATR center and the reputation of the public institution*.

**Hypothesis** **9** **(H9).** *The perceived value of the ATR center’s services has a mediation effect between perceived justice and the reputation of the public institution* **.**

**Figure 4:**
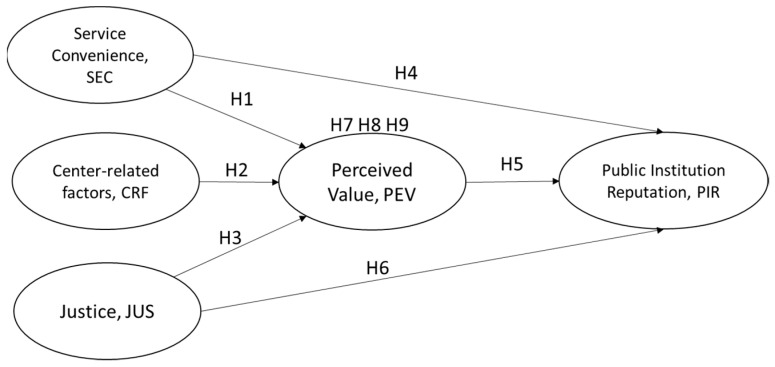
Research Model.

This study was conducted to examine the perceptions of users of assistive devices or their caregivers on the use of the ATR center’s services and to examine the influencing factors. In this study, convenience of service, factors related to the ATR center, justice and perceived value were used as independent variables, where perceived value played the role of a mediating variable and reputation of the public institution was a dependent variable. The structure of this study was developed from the literature as shown in Figure 4.

## 3. Research Design

### 3.1. Research Subjects and Data Collection

The total population of Yunlin County is 674,622, and there are 134,439 people over 65 years of age, which is 19.34% of the elderly population. The number of people with disabilities is 50,499, accounting for 7.48% of the total population. This study was conducted to investigate the feelings of the users of assistive devices after receiving services from the ATR center, such as assistive device evaluation, repair or assistive device loan services. Therefore, the target population was assistive device users or their caregivers within two years. Then, a questionnaire was used to conduct the survey, and volunteers assisted in distributing paper copies and online e-questionnaires. Research data was collected in various townships in Yunlin County through organizations for the physically and mentally challenged, long-term care institutions, ATR centers and service bases, and aids manufacturers. A total of 320 questionnaires were collected during the distribution period from 1 May 2021 to 20 September 2021, after removing invalid questionnaires, the total number of valid questionnaires was 318.

### 3.2. Measurement Instrument

The research variables in this study include: the basic background of aids users, service convenience, factors related to the ART center, justice, perceived value, and the reputation of the public institution. In order to make the concepts between the research variables specific, the operational definitions of each variable are described below.

#### 3.2.1. Basic Background of the Individual User of the Aids

This study focuses on the public’s perception of the overall service after receiving the service of the ATR center. Therefore, basic personal information would be measured in the first part of the survey. It contains seven items: gender, age, physical and mental disability qualification, type of physical and mental disability, level of physical and mental disability, type of service received by the ATR center, and the identity of the respondent (Table 1).

#### 3.2.2. Measurement Questionnaire Design

The questions were measured on a seven-point Likert scale, ranging from strongly disagree (1) to strongly agree (7), with higher scores indicating higher levels of agreement with the study variables. After the design of the questionnaire, experts were invited to review the questions and give their opinions. The design of the questionnaire for each structure is described as follows.

1. Service Convenience

This study on the construct of service convenience refers to Roya, Shekharb, Lassarc, and Chend [52], conducting research on customer behavior to evaluate service convenience, justice, and service quality, etc. The study classifies service convenience into the components of ease of access to services, ease of decision to purchase, and ease of seeking services. The CR reliability value of its construct is 0.88. Five questions were used in this study. The original questions for the service convenience section are “Service provider was available when I needed them”, “Service provider is accessible through various ways (online, telephone, & in person)”, “Hours of operation were convenient”, “Service provider offers convenient locations”, and “Information received from the service provider made it easy to choose what to buy”. A total of 5 questions were designed with reference to the convenience of accessing services at the ATR center.

2. The ATR Center Related Factors

This study refers to Guang-Xu Wang’s article [53] for the composition of factors related to the ATR center. The study examined the association between the quality of community presence services and the degree of successful aging enhancement. Referring to the moderating effect of the government’s role perception, the questions “I think the activity space inside the institution is well planned.” and “I think the overall facilities of the institution meet the needs.” were revised, and a total of 2 questions were designed. In addition, the research questions “This brand makes me feel reliable.”, “This brand makes me feel professional.”, and “This brand makes me feel friendly.” were modified by referring to the research conducted by Hsiang-Hsi Liu Teng-Tsai Tu and Chien-Sheng Lo [54] which examined the impact of brand image on consumer satisfaction and loyalty from the viewpoint of relationship value and relationship quality in Taiwan’s notebook computer industry. Three questions were designed.

3. Justice

The justice dimension refers to the study by Chao-Min Chiu, Chao-Sheng Chiu, Hae-Ching Chang [29]. Their study focused on the effects of learners’ web-based learning continuance intentions, examining the integrated influence of fairness and quality on learners’ satisfaction. The items “The instructor showed concerns for my rights as a student.”, “The instructor treated me with dignity and respect.”, and “The instructor provided me with timely feedback to my questions.” were modified. This study also references Chao-Min Chiu, Szu-Yuan Sun, Pei-Chen Sun, Teresa L. Ju’s study [29]. The items “The instructor provided opportunities to appeal or challenge the grading decisions.”, and “The instructor generated standard so the grading decisions could be made with consistency.” were referenced. There are total five items.

4. Perceived Value

This questionnaire is based on Ivan K. W. Lai’s [55] survey question “The overall value of eating at this HKST restaurant was high.”, “The eating experience on this HKST restaurant was worth the money.” and Shih-Chih Chen, Chieh-Peng Linb’s study [56], the impact of customer experience and perceived value on sustainable social relationships in tribal communities. The items “The blogs offer me various options of products or services.”, “The information I will have acquired on the blogs will enable me to do my life/job better.” were revised. A total of 4 questions were designe.

5. Public Institution Reputation

This study focuses on the public institution reputation with reference to the items of Heetae Yang, Jieun Yu, Hangjung Zo, and Munkee Choi’s research [57], “I always have great confidence in the low-cost airline’s service.” and “I always have positive impressions of low-cost airline. These two questions focused on the impact of service quality and price perception on word-of-mouth and revisit intent. Moreover, Yu-Shan Chen and Stanley Y.J. Huang’s items [58] were “I feel the image of the e-learning instructors (or teachers) is good.” and “I feel the e-learning provider [company name] is well known”. These two questions were revised and translated. The experts were asked to review whether the translations matched the meaning of the questions. A total of five questions were designed after the revision.

### 3.3. Data Analysis

The data analysis in this study will be conducted in three stages: descriptive analysis, measurement model validation, and structural equation modeling, in which descriptive statistics includes statistical analysis of demographic variables and calculation of the mean and standard deviation of each component to understand the concentration of each variable [59]. Then, Confirmatory Factor Analysis (CFA) will be conducted to confirm the reliability of the questions, which includes: Composite Reliability to measure the internal consistency of the variables, Convergent Validity and Discriminant validity. In the third stage, a Structural Equation Model (SEM) analysis will be implemented to examine the fit of the model and then to validate the hypotheses of this research framework by statistic software AMOS (SPSS Inc., Chicago, IL, USA).

## 4. Results

### 4.1. Descriptive Statistical Analysis

Most of the respondents were female, accounting for 174 (60.4%), aged 51–60, accounting for 64 (22.2%), and “no” physical or mental disabilities for 167 (58.0%). Then, 121 (42.0%) had physical or mental disabilities, including 82 (28.5%) in category 7 (physical disabilities); and 203 (70.5%) were caregivers or family members. The data are shown in Table 2.

### 4.2. Types of Assistive Device Services and Locations

The following is a survey of the types of assistive device services received by the respondents and the locations where they know which types of assistive device services are currently provided. The survey was conducted in a multiple-choice format. The purpose is to figure out the understanding of the target users of the assistant aids to the existing services. The relevant statistics are as follows.

#### 4.2.1. Service Received from the ATR Center

There are eight kinds of services received from the ART center, and the frequency distribution table shows that “consulting”, “lending” and “assessment” were the top three kinds of services received by the center (Table 3). The number of responses for “consulting” is 139, the percentage is 21.5%, the observation percentage is 48.3%, The center service option was a multiple choice question, so 43.8% (139 people) of respondents came to the center for the purpose of consulting. The number of responses for “lending” was 127, the percentage was 19.6%, and the observation percentage was 44.1%, Then, the number of responses for “assessment” is 126, the percentage is 19.5%, the observation percentage is 43.8%,

#### 4.2.2. Types of Assistive Device Services Available in the County

In Taiwan, in addition to the ATR centers that provide a complete range of services, there are also assistive technology service bases that provide services with certain frequency (e.g., once a week) in towns far from the ATR centers. Service base provides services such as assessment, repair and rental of auxiliary equipment. Moreover, smaller assistive technology convenience stations are located in remote areas, providing only simple rental and return services for assistive devices.

The types and location of assistive device services is a multiple question. Statistically, what service location and types the test subjects know are as follows (Table 4). There are three types of assistant aids service locations in Yunlin County. As shown in the frequency distribution table, “1. the ATR centers (Douliu and Beigang)” were answered 243 times, with a percentage of 63.6% and an observation percentage of 87.7%, “2. assistant aids service bases (Tukou Health Center, Taisi Health Center, Yunji Hospital, Beima Hospital, Rouser Hospital, and Huwei Complex)” were answered 103 times, with a percentage of 27.0% and an observation percentage of 37.2%, and “3. convenient stations for assistant aids services (township health center)” was 36, the percentage was 9.4%, and the percentage of observations was 13.0%.

### 4.3. Convergent Validity

Anderson and Gerbing [59] suggested that Structural Equation Modeling (SEM) should be implemented in two stages. The first stage is to validate the measurement by Confirmatory Factor Analysis (CFA) to verify the reliability and validity of the measurement. In the second stage, maximum likelihood estimation (MLE) is used to determine the significance of the study hypotheses. As Table 5, all standardized factor loadings of questions are from 0.668 to 0.955 falling into a reasonable range. This demonstrate all questions have convergent validity. All the composite reliability of the constructs ranging from 0.913 to 0.964, exceed 0.7 recommended by Nunnally and Bernstein [60] indicating all constructs have internal consistency. Lastly, all average variance extracted (AVE) ranging from 0.677 to 0.868, exceed 0.5 suggested by Hair, Anderson, Tatham, and Black [61] and Fornell and Larcker [62] showing all constructs have adequate convergent validity.

### 4.4. Discriminant Validity

Fornell and Larcker [62] suggested that the square root of the average variance extracted (AVE) for each construct be used to compare the correlation coefficients of other constructs to examine the discriminant validity among the constructs in the study model. As Table 6, the bold numbers in the diagonal direction represent the square roots of AVEs. Because all the numbers in the diagonal direction are greater than the off-diagonal numbers, discriminant validity appears to be satisfactory for all constructs.

### 4.5. Model Fit

In this study, the model fit metrics were used as a blueprint for the applied model fit analysis by referring to the 194 SSCI papers studied in Jackson, Gillaspy, and Purc-Stephenson [63], and the nine most widely used fit metrics were used to report the papers. The results of this study were found to be an acceptable model by passing each of the suitability indicators (Table 7).

### 4.6. Path Analysis

**Figure 5:**
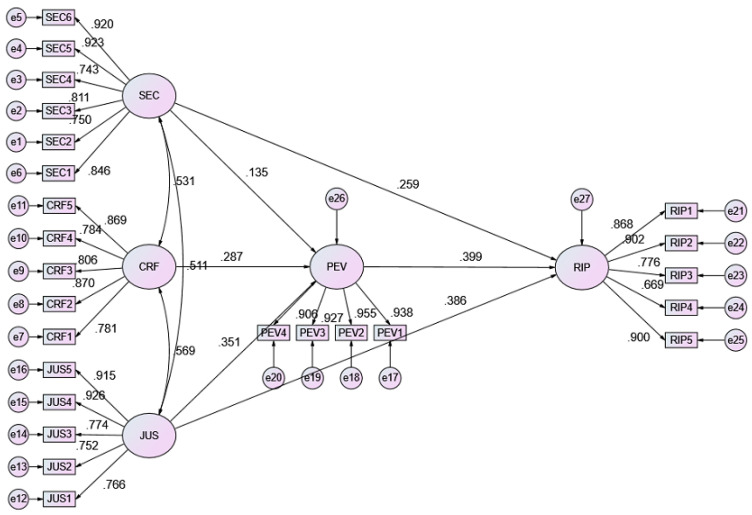
SEM model analysis.

The Table 8 shows the results of path coefficients. Service convenience (*b* = 0.150, *p* = 0.027), center-related factors (*b* = 0.284, *p* < 0.001), and justice (*b* = 0.388, *p* < 0.001) significantly impact perceived value. Service convenience (*b* = 0.234, *p* < 0.001), justice (*b* = 0.349, *p* < 0.001) and perceived value (*b* = 0.326, *p* < 0.001) significantly impact public institution reputation. The results support the research question regarding the validity of the research model. 42.8% of perceived value can be explained by service convenience, center-related factors, and justice constructs. 75.4% of public institution reputation can be explained by service convenience, justice and perceived value constructs. Figure 5 displays SEM model analysis. In addition, statistical significance is easily affected by sample size, and the larger the sample amounts are, the easier it is to obtain the significance level of the regression coefficient. In view of this, the effect size can be used to assist in the interpretation of statistical significance. The criterion for determining the effect size (ES) is low when f^2^ = 0.02~0.15, medium when f^2^ = 0.15~0.35, and high when f^2^ > 0.35. Service Convenience (SEC) for Perceived Value (PEV) f^2^ = 0.012 does not meet the criteria; Center-related Factors (CRF) for Perceived Value (PEV), f^2^ = 0.070 has a low effect size; Justice (JUS) has a low effect size for Perceived Value (PEV), f^2^ = 0.108; Service Convenience (SEC) has a medium effect size for Public Institution Reputation (RIP), f^2^ = 0.154; Justice (JUS) has a medium effect size for Public Institution Reputation (RIP) f^2^ = 0.313 has a medium effect size; Perceived Value (PEV) has a medium effect size for Public Institution Reputation (RIP) f^2^ = 0.325.

### 4.7. Mediation Effect Analysis

As shown in Table 9, the total effect SEC→RIP, bias-corrected confidence interval (CI) does not include 0 (CI of SEC→RIP = [0.027 0.853]). The existence of total effect was supported. The total indirect effect SEC→RIP, *p* > 0.05, bias-corrected confidence interval (CI) does include 0 (CI of SEC→PEV→RIP = [−0.008 0.515]). The existence of total indirect effect was not supported. It was not necessary to test mediation effect. The total effect CRF→RIP, *p* > 0.05, bias-corrected confidence interval (CI) does include 0 (CI of CRF→PEV→RIP = [0 0.716]). The existence of total effect was not supported. It was not necessary to test mediation effect. The total indirect effect CRF→RIP, *p* > 0.05, bias-corrected confidence interval (CI) does include 0 (CI of CRF→RIP = [0 0.716]). The existence of total indirect effect was not supported. It was not necessary to test mediation effect. The total effect JUS→RIP, bias-corrected confidence interval (CI) does not include 0 (CI of JUS→RIP = [0.125 1.12]). The existence of total effect was supported. The total indirect effect JUS→PEV→RIP, bias-corrected confidence interval (CI) does not include 0 (CI of JUS→RIP = [0.026 0.838]). The existence of total indirect effect was supported.

## 5. Conclusions and Discussion

This study examines the perceptions of the users and their caregivers of the government-established assistive device centers in Taiwan and examines the impact on the reputation of the public institution to serve as a reference for the design of government-implemented assistive device services for the elderly and the physically and mentally disabled. The research framework and hypotheses were developed by examining the factors of “service convenience”, “center-related factors”, “justice”, and “perceived value” and using “perceived value” as a mediating variable. The data were collected through a questionnaire survey, and the structural equation model was used to test the model and verify the hypotheses.

### 5.1. Academic Contributions

In the past 20 years, Taiwan has been actively promoting assistive device services for people with disabilities (such as the elderly and the physically and mentally challenged). In addition to the regular evaluation of the ATR centers established in each county and city to improve the quality of service, a budget has been allocated to hire additional professional manpower. By setting up many service bases, users with limited mobility can enjoy convenient access to assistive device services. However, in the overall service design process, the perception of users of assistive devices and the related influencing factors when receiving services are less explored. There are no studies on the influence of the process of assistant aid services on the prestige of the government.

Therefore, this study attempts to examine its impact on the government’s reputation in terms of service convenience, factors related to the ATR center itself, justice, and the perceived value. The results are obtained as follows.

#### 5.1.1. Impact of Service Convenience, Center-Related Factors, and Justice on Perceived Value

The results of the study revealed that convenience of service, factors related to the ART center, and justice all had a significant positive effect on perceived value, which is similar to previous studies [15,22]. In addition, for the items of service convenience, such as multiple channels to obtain aids and sufficient information to choose aids, etc.; or the items of center related factor, which includes internal space planning, overall equipment and services that make people feel professional and reliable, etc.; or the question of “justice”, such as the staff of the aids center giving sufficient respect to aids users and giving consistent evaluation standards, etc. The average score of these items were 6.1 or higher. The convenience of the service, the center related factors, and the justice of receiving the service are all important to the users of assistive devices. There is a consensus on enhancing the perceived value of the service. In the path analysis of the perceived value of justice, the non-standardized regression coefficient reached 0.388, indicating that the perceived value of justice affects the users of assistive devices with a very high proportion, even much higher than the 0.150 of convenience of service.

#### 5.1.2. Impact of Service Convenience, Perceived Value and Justice on Public Institution Reputation

In terms of the effects of service convenience, justice and perceived value on the reputation of public institution, the results of this study showed that service convenience, justice and perceived value all have positive effects on the reputation of public institution, which is similar to the results of previous studies [44,47]. Moreover, the R2 value reaches 0.754, the explanation power is very high. In this regard, justice has a higher degree of influence than other components, which shows that the relatively disadvantaged groups, such as those in need of assistive devices, value the justice of treatment provided by the service side more than the general population [64]. The research data also showed that when people in need of assistive devices seek assistive devices-related services, they have an average score of 6.434 or higher in terms of their psychological feelings about their rights, whether they are respected when receiving the services, and even whether they can respond in a timely manner during consultation. It also has an impact on the reputation of the public institution.

In addition, from the study results, it was found that it would be more convenient to apply for the related assistant aids services provided by the government, for example, there are many channels to contact the services, convenient service locations or easy access to relevant information, especially when the need for assistant aids is often urgent or pressing, for example, when a wheelchair breaks down and cannot be repaired, or when a sudden stroke attack requires assistance from the ATR center. When a wheelchair or homecare bed is needed, the ART center can help. This kind of service that meets the needs of the public in a timely manner has a very positive impact on enhancing the reputation of public institutions. In particular, although the mean score of “the ATR center is well known” is 6.072, it has the highest standard deviation (SD = 1.074) among all the questions. It also shows that there is still room for the public institution to work on how to make the ATR center known to those who need it and what services it provides.

Users of assistive devices often have various needs for assistive device services, including purchasing assistive devices that require assessment and consultation by the ATR center, repairing assistive devices that are faulty, or trying out assistive devices on display at the ATR center…etc. Therefore, users of assistive devices expect that such permanent assistive device service institution of government departments can provide a wide range of service options and a more valuable service experience, and even improve their daily lives with the assistance of the ATR center. This study also scored average over 6.399 on the perceived value items, indicating that users consider a high level of perceived value necessary after receiving the service.

#### 5.1.3. Impact of Service Convenience, ATR Center-Related Factors, and Justice on Perceived Value on the Reputation of the Public Institution

The results of the study show that both ATR center-related factors and justice affect perceived value, and perceived value also significantly and positively affects public institution reputation, i.e., ATR center-related factors and justice indirectly affect public institution reputation, and perceived value has a mediating effect. This result is similar to the results of previous studies [50,51]. The researcher thought that the service convenience might positively affect the perceived value and indirectly enhance the public reputation, but this is not true. It is possible that users of assistive devices rarely receive ATR services, and only when there is an urgent need, such as a broken assistive device requiring repair or a government subsidy application, will they seek the services of the ATR center. Therefore, regardless of the convenience of the service, the necessity of the service does not affect the perceived value of the service received and indirectly affects the reputation of the public entity. On the contrary, center related factors, such as well-planned internal space or friendly staff, as well as justice factors such as service recipients feeling respected or service staff caring about their rights, allow service users to feel the perceived value of the service, which in turn positively affects the reputation of public institutions. It can be seen that when providing auxiliary aids services, the equipment and space planning of ART centers should be improved, and the training of center personnel should be enhanced so that the public can feel friendly, reliable and professional, in order to enhance the perceived value of ART services and further increase the reputation of the institution.

### 5.2. Practical Suggestions

The World Health Organization (WHO) has proposed the Global Strategy and Plan of Action on Ageing and Health [1] to address the global trend of population aging. It recognizes that if the functional capacity of the elderly is maintained, and if a supportive and friendly environment is provided, the overall health status of the elderly can be enhanced so that they can continue to participate in society and improve their health status and social well-being. Therefore, the appropriate use of assistive technology devices and the provision of a Barrier-free environment can provide more support to the elderly and increase opportunities for social participation. Therefore, the appropriate use of assistive technology devices and the provision of a Barrier-free environment can provide more support to the elderly and increase opportunities for social participation. The appropriate use of assistive technology devices and the provision of a barrier-free environment can provide more support to the elderly and increase opportunities for social participation. At the same time, such social design can be achieved through a user-centered approach to service design, and through designers’ active engagement in contextual and collaborative learning to make the overall senior service work [65].

International Organization for Standardization (ISO) considers assistive products as “assistive living convenience products”, which not only have assistive devices specifically designed for people with legal physical and mental disabilities, but also refer to products and technologies applied by all people in their daily lives. products and technologies [66]. Therefore, the application of Assistive Technology is to alleviate the difficulties of the disabled and to improve or maintain the physical function, structure, promote activity and participation, or facilitate the care of their caregivers [67].

In this study, the target population of the ATR center in Yunlin County is facing the double dilemma of a large growth of the population in need of auxiliary aids and a shortage of caregivers as the overall population structure is rapidly moving towards an ultra-high-age society. The use of assistive devices can reduce the caregiver’s burden, enhance the care recipient’s ability to move independently, and delay disability. Therefore, Yunlin County has been actively promoting the provision of professional, comprehensive and convenient assistive device services to users and caregivers. In addition, most of the responders were caregiver or family members (70.5%). The findings of the study suggest that government departments should place more emphasis on the satisfaction of caregivers or family members of aids users with the justice, convenience and related factors of the aids services. This will enhance the reputation of the public institutions. The results of this study suggest that the following three directions should be promoted in the future to enhance the quality of public institutions in promoting assistive device services:

#### 5.2.1. Pay Attention to the Justice of the Service for the Demand of Auxiliary Aids

About the justice construct of the study, it was found that the perception of justice in receiving services had the highest impact on improving the reputation of the public institution. Therefore, in addition to providing professional services to the clients, the ATR center should also pay more attention to the rights of the clients, listen to their needs and provide respectful services, and establish a mechanism to complain about service dissatisfaction and give the clients an opportunity to express their opinions. In addition, professional evaluation training with consistent standards should be conducted, such as regular case studies and a supervisory mechanism to develop evaluation reports, so as to enhance the perception of justice of service recipients, which will not only increase the perceived value of the service but also help to enhance the reputation. Finally, on-site staff should be responsive and approachable, always on the lookout for unattended clients and proactive in providing service. This finding is also consistent with Rui Miaoa’s [68] case study on healthcare in China, which found that the four value dimensions of access to healthcare, including economic value, health value, fair value, and supplemental value, had a significant impact on patient satisfaction and patient loyalty.

#### 5.2.2. Thinking about How to Increase Service Convenience and Enhance the Perceived Value of Services

This study found that an increase in service convenience would directly and positively affect the reputation of the public institution. For example, opening weekend or nighttime service hours for family members’ convenience, providing multiple channels to access the services of the center, or providing convenient service locations will help improve the overall service convenience. In addition, by improving the equipment and planning space of the ATR center, as well as taking into account the perceived justice of the service, or making the service recipients aware of the variety of service promotion in the ATR center, the perceived value of the service can be increased, allowing the service recipients to have a better service experience and indirectly enhancing the overall reputation. From the findings of a case study by Álvarez Cano, Ana Milena [69] et al. on the evaluation of perceived service value in sporting events. It is clear that the infrastructure of sporting events, as well as customer service, contributes to the perceived value and satisfaction of fans.

#### 5.2.3. Strengthening the Center Related Factors

In the process of service design, each touchpoint is an opportunity to shape the feelings of each service user. Therefore, whether it is the service process, the spatial design of the service experience or the feeling of the equipment experience, all of them will have an evaluation on the feeling of the service, which in turn will affect the perceived value of the service recipients. Therefore, in the future, the ATR center should pay more attention to the overall experience of the space, whether it is the spatial design, the information of the on-site auxiliary equipment, or the spatial aesthetics in public institutions. At the same time, the professional image and friendly service of the staff should be strengthened so that the service recipients can have a better feeling about the service experience, which will increase the perceived value and indirectly affect the reputation of the public institution.

At the same time, by integrating the training and organizational strategies of the staff of the centers, the staff will learn more about the organizational knowledge of the centers, which will lead to better service delivery [70]. A case study conducted in a public hospital in the city of Kocaeli, Turkey [71] found that healthcare workers’ perceptions of their organization’s prestige can also contribute to increased work engagement, which in turn increased customer satisfaction.

### 5.3. Research Limitations and Future Developments

The sampling process of this study coincided with the serious spread of COVID-19 in Taiwan, which made questionnaire collection extremely difficult. The main sampling targets were mainly the clients who went to the service locations (e.g., ATR centers, service bases), so that only those who were familiar with the services and service locations of the ATR centers were adopted for the study. Those who were unable to go out or lacked information about the ATR centers were not consulted. In the future, if there is further research, the researchers can go deeper into the community to conduct further research. In addition, this study focuses on the target population of the ATR center in Yunlin County, because the population composition of Yunlin County tends to be older. The transportation and economic income of the general public is not as high as that of metropolitan cities such as Taipei City or Kaohsiung City. Future studies should be conducted in other cities with different backgrounds to understand more comprehensive service perceptions and to provide different service designs.

In addition, as there is relatively little research and discussion on assistive technology services, it is suggested that a real time case study approach could be considered in order to obtain more practical research results. The real time case research method is originally an innovative teaching practice that uses various technologies to create new types of teaching and learning, and uses them to discuss the benefits of (1) real-time interactivity and (2) extended coverage [72]. Such case studies are mostly applied in business market analysis and in the education system. For example, the discussion of the effectiveness of a training intervention [73]. Moreover, a big data based method for pass rates optimization in mathematics for lower division courses [74]. Or, case studies on microentrepreneurs and exploring their implications for higher education [75].

## Figures and Tables

**Table 1:** Demographic data.

Variable	Value Label
Gender	Male
	Female
Age	21–30 years old
	31–40 years old
	41–50 years old
	51–60 years old
	61–70 years old
	71–80 years old
	81 years or above
Physical and Mental Disorders Qualifications	People with disabilities
	“No” physical or mental disability eligibility-“Yes” used long term care services
	“No” physical or mental disability eligibility-“No” use of long-term care services
Physical and Mental Disorders Category	Category 1–Category 8
Physical and mental impairment level	Others
	“No” physical or mental disability qualification
	Mild
	Moderate
	Severe or above
	No physical or mental disability qualification
Identity of the respondent	Aid device users themselves
	Caregiver or family member
	Others

**Table 2:** Frequency distribution table.

Variable	Value Label	Frequency	Percent
Gender	Male	114	39.6
	Female	174	60.4
Age	21–30 years old	35	12.2
	31–40 years old	33	11.5
	41–50 years old	50	17.4
	51–60 years old	64	22.2
	61–70 years old	32	11.1
	71–80 years old	33	11.5
	81 years or above	41	14.2
Physical and Mental Disorders Qualifications	People with disabilities	121	42.0
	“No” physical or mental disability eligibility-“Yes” used long term care services	57	19.8
	“No” physical or mental disability eligibility-“No” use of long-term care services	110	38.2
Physical and Mental Disorders Category	Category 7	82	28.5
Physical and mental impairment level	Others	39	13.5
	“No” physical or mental disability qualification	167	58.0
	Mild	31	10.8
	Moderate	37	12.8
	Severe or above	53	18.4
	No physical or mental disability qualification	167	58.0
Identity of the respondent	Aid device users themselves	75	26.0
	Caregiver or family member	203	70.5
	Others	10	3.5

**Table 3:** Service received from the ART center.

Service	N	Percentage	Observation Percentage
1. Consulting	139	21.5%	48.3%
2. Lending	127	19.6%	44.1%
3. Assessment	126	19.5%	43.8%
4. Subsidy Application	114	17.6%	39.6%
5. Repairing	77	11.9%	26.7%
6. Recycling	27	4.2%	9.4%
7. Fitting & Training	25	3.9%	8.7%
8. Others	12	1.9%	4.2%
Total	647	100.0%	224.7%

Note: N = Number of respondents answering the question option.

**Table 4:** Types and locations of assistive device services in Yunlin County.

Service Type and Location	N	Percentage	Observation Percentage
1. ATR centers (Douliu and Beigang)	243	63.6%	87.7%
2. Assistive technology service bases (Tukou Health Center, Taisi Health Center, Yunji Hospital, Beima Hospital, Rouser Hospital, and Huwei Complex)	103	27.0%	37.2%
3. Assistive technology convenience stations (ownship health center)	36	9.4%	13.0%
Total	382	100.0%	137.9%

Note: N = Number of respondents answering the question option.

**Table 5:** Results for the measurement model.

Construct	Item	Significance of Estimated Parameters				Item Reliability		Construct Reliability	Convergence Validity
		Unstd.	S.E.	Unstd./S.E.	*p*-Value	Std.	SMC	CR	AVE
SEC	SEC1	1.000				0.846	0.716	0.932	0.698
	SEC2	0.979	0.064	15.276	0.000	0.751	0.564		
	SEC3	1.161	0.068	17.138	0.000	0.811	0.658		
	SEC4	1.114	0.075	14.926	0.000	0.743	0.552		
	SEC5	1.128	0.052	21.504	0.000	0.923	0.852		
	SEC6	1.105	0.052	21.396	0.000	0.920	0.846		
CRF	CRF1	1.000				0.780	0.608	0.913	0.677
	CRF2	0.989	0.059	16.735	0.000	0.869	0.755		
	CRF3	0.936	0.065	14.294	0.000	0.808	0.653		
	CRF4	0.911	0.067	13.655	0.000	0.783	0.613		
	CRF5	0.887	0.058	15.341	0.000	0.869	0.755		
JUS	JUS1	1.000				0.766	0.587	0.917	0.690
	JUS2	0.981	0.072	13.647	0.000	0.752	0.566		
	JUS3	1.009	0.072	14.051	0.000	0.774	0.599		
	JUS4	1.148	0.067	17.214	0.000	0.927	0.859		
	JUS5	1.105	0.065	17.014	0.000	0.915	0.837		
PEV	PEV1	1.000				0.938	0.880	0.964	0.868
	PEV2	1.010	0.030	33.848	0.000	0.955	0.912		
	PEV3	0.945	0.031	30.037	0.000	0.927	0.859		
	PEV4	0.942	0.034	27.787	0.000	0.907	0.823		
RIP	RIP1	1.000				0.866	0.750	0.915	0.685
	RIP2	0.978	0.045	21.763	0.000	0.904	0.817		
	RIP3	1.005	0.062	16.317	0.000	0.775	0.601		
	RIP4	1.086	0.084	12.853	0.000	0.668	0.446		
	RIP5	1.018	0.048	21.355	0.000	0.900	0.810		

Note: Unstd. = Unstandardized factor loadings; Std = Standardized factor loadings; SMC = Square Multiple Correlations; CR = Composite Reliability; AVE = Average Variance Extracted; SEC = Service Convenience; CRF = Center-related Factors; JUS = Justice; PEV = Perceived Value; RIP = Public Institution Reputation.

**Table 6:** Discriminant validity for the measurement model.

	AVE	SEC	CRF	JUS	PEV	RIP
SEC	0.698	**0.835**				
CRF	0.677	0.531	**0.823**			
JUS	0.690	0.511	0.569	**0.831**		
PEV	0.868	0.467	0.559	0.583	**0.932**	
RIP	0.685	0.642	0.580	0.751	0.745	**0.828**

Note: The items on the diagonal on bold represent the square roots of the AVE; off-diagonal elements are the correlation estimates. SEC = Service Convenience; CRF = Center-related Factors; JUS = Justice; PEV = Perceived Value; RIP = Public Institution Reputation. The bold numbers in the diagonal direction represent the square roots of AVEs.

**Table 7:** Model fit.

Fit Indices	Criteria	Model Fit	Results
Chi-square		715.753	
Degree of freedom		266	
CFI	>0.9	0.936	Pass
RMSEA	<0.08	0.077	Pass
TLI	>0.9	0.927	Pass
GFI	>0.9	0.902	Pass
NFI	>0.9	0.902	Pass
χ^2^/*df*	<3	2.688	Pass
AGFI	>0.8	0.874	Pass

**Table 8:** Regression coefficient.

DV	IV	Unstd	S.E.	Unstd./S.E.	*p*-Value	Std.	R^2^	f^2^
PEV	SEC	0.150	0.068	2.217	0.027	0.135	0.428	0.012
	CRF	0.284	0.066	4.278	0.000	0.287		0.070
	FAI	0.388	0.073	5.322	0.000	0.351		0.108
POR	SEC	0.234	0.041	5.772	0.000	0.259	0.754	0.154
	FAI	0.349	0.048	7.350	0.000	0.386		0.313
	PEV	0.326	0.039	8.324	0.000	0.399		0.325

Note: SEC = Service Convenience; CRF = Center-related Factors; JUS = Justice; PEV = Perceived Value; RIP = Public Institution Reputation

**Table 9:** The analysis of indirect effects.

Effect	Point Estimate	Bootstrap 1000 Times			
		Bias-Corrected 95%			
		S.E.	Z-Value	Lower Bound	Upper Bound
Total effect					
SEC→RIP	0.283	0.223	1.269	0.027	0.853
Total indirect effect					
SEC→PEV→RIP	0.049	0.097	0.501	−0.008	0.515
Direct effect					
SEC→RIP	0.234	0.189	1.241	0.031	0.749
Total effect					
CRF→RIP	0.093	0.172	0.54	0	0.716
Total indirect effect					
CRF→PEV→RIP	0.093	0.172	0.54	0	0.716
Total effect					
JUS→RIP	0.476	0.327	1.454	0.125	1.12
Total indirect effect					
JUS→PEV→RIP	0.127	0.155	0.817	0.026	0.838
Direct effect					
JUS→RIP	0.349	0.329	1.062	0.031	1.08

Note: SEC = Service Convenience; CRF = Center-related Factors; JUS = Justice; PEV = Perceived Value; RIP = Public Institution Reputation.

## Data Availability

Not applicable.
